# Massachusetts Dental Society

**Published:** 1867-07

**Authors:** Edward N. Harris

**Affiliations:** Boston.


### CORRESPONDENCE.

---

### *Massachusetts Dental Society.*

Reported by EDWARD N. HARRIS, D. D. S., of Boston.

THE fourth Annual Meeting of the Massachusetts Dental Society, was held in Boston, on the 23d of May, at their Hall, No. 12 Temple Place, the President, N. C. Keep, M. D., of Boston, in the chair.

There was a full attendance of members from Boston and different parts of the State. The first two hours of the session, from 9 until 11½ o'clock, were occupied with clinics, by Drs. G. T. Moffatt, S. J. McDougall, T. B. Hitchcock and J. T. Codman, consisting of complicated operations of filling teeth. These exhibited a high degree of skill, and were witnessed with no little interest.

Several interesting specimens of morbid anatomy and of operative and mechanical dentistry were presented to the museum of the Society.

The annual reports of the Recording Secretary, Librarian, Treasurer, and Executive Committee were submitted and accepted. The report of the Treasurer showed that the receipts for the year were $368.75; expenditures $142.-30; leaving a balance on hand of $225. The report of the Librarian showed that a good foundation has been laid for a library and museum.

Dr. L. D. Shepard, of Salem, reported progress in regard to the establishment of the proposed New England Dental Magazine, of which Dr. T. H. Chandler, of Boston, has been appointed one of the editors.

The Society then elected the following list of officers for the ensuing year: President, E. G. Leach, D. D. S., of Boston; Vice Presidents, H. F. Bishop, D. D. S., of Worcester, and E. N. Harris, D. D. S., of Boston; Corresponding Secretary, E. C. Rolfe, M. D., of Boston; Recording Secretary, J. T. Codman, M. D., of Boston; Librarian, G. T. Moffatt, M. D., of Boston; Treasurer, S. J. McDougall, M. D., of Boston; Executive Committee, T. H. Chandler, A. M., of Boston, T. H. Hitchcock, M. D., of Boston, G. T. Moffatt, M. D., of Boston, Dr. Edmund Blake, of Boston, and L. D. Shepard, D. D. S., of Salem.

Dr. Thomas H. Chandler was chosen orator for 1868, and Dr. S. J. McDougal as substitute.

The following persons were chosen delegates to the American Dental Association in Cincinnati on the last Tuesday of July: Drs. D. G. Williams, J. T. Codman, E. Blake, J. Thompson, C. F. Horne, W. S. Miller, T. H. Chandler, D. G. Harrington, A. Brown, U. K. Mayo, C. Whitechurch, D. W. Leach, A. Papineau and B. T. Currier.

Dr. E. G. Leach, on taking the chair, paid a merited tribute to the efficiency and services of the venerable retiring President, and expressed his own thanks for the honor conferred upon himself.

Henry F. Bishop, D. D. S., of Worcester, then delivered the annual address, which was a very able and excellent dissertation and was received with generous applause. At its close a resolution was unanimously passed, tendering the thanks of the Society to Dr. Bishop, for his most excellent address, and requesting a copy for publication in the proposed New England Dental Journal, and also two hundred and fifty copies to be printed in pamphlet form for distribution among the members of the Society. A

copy of this address has also been requested for publication in the American Journal of Dental Science, and will probably appear in a future number of that valuable Journal.

At half past three o'clock, the Society adjourned to the Tremont House, where the Anniversary dinner was provided. The dinner was served in an excellent manner, and when appetites had been appeased, several brief and pleasant after-dinner speeches were made, after which, the Society re-assembled in one of the parlors of the Tremont House, where the remainder of the evening was passed in an interesting and profitable discussion on the best methods of filling teeth.

This Society was organized in 1864, incorporated in 1865. It holds regular monthly meetings, on the second Tuesday evenings of each month, which are devoted to discussions in the different departments of dental practice, the reading of essays, and the presentation and examination of specimens of operative and mechanical dentistry.

